# Assessing Multiple Risks in Regulating Reservoirs: Perspectives on Heavy Metal Contamination

**DOI:** 10.3390/toxics13090762

**Published:** 2025-09-08

**Authors:** Hui Zhou, Zhiping Li, Anming Wang, Jiawei Zhu, Zongyuan Han, Yalin Zhang, Dongdong Chen

**Affiliations:** 1 College of Geosciences and Engineering, North China University of Water Resources and Electric Power, Zhengzhou 450046, China; zhouhui@ncwu.edu.cn (H.Z.); zhujiawei1024@163.com (J.Z.); x20241020227@stu.ncwu.edu.cn (Z.H.); zhang671431@163.com (Y.Z.); cdd341323@163.com (D.C.); 2 Henan Vocational College of Water Conservancy and Environment, Zhengzhou 450046, China

**Keywords:** heavy metal, Dongping Lake, ecological-risk-regulating reservoir, surface sediment, water

## Abstract

As the terminal reservoir of the South-to-North Water Diversion’s Eastern Route, Dongping Lake is critical for safeguarding the northern water supply. Analysis of 33 water–sediment sites revealed the following. (1) Waterborne heavy metals (HMs) below WHO limits, confirming the good water quality. (2) Sediment HM enrichment exceeding background levels, with Cd posing high ecological risk (mean *E_r_* = 135), and moderate overall pollution. (3) Speciation showed V, Cr, Co, Ni, Cu, Zn, and Pb predominantly in residual fractions, while Cd exhibited high bioavailability and Pb was in reducible state. Ecological risk assessment indicated that V and Cr tend not to cause environmental pollution; Co, Ni, Cu, Zn, and Pb only cause slight pollution; and Cd causes serious point-source pollution. The carcinogenic risk of surface sediments to children is not negligible. (4) Source apportionment identified industrial emissions as the primary HM contributors, with Cd deriving from agricultural runoff (phosphate fertilizers) and industrial discharges. This study offers valuable baseline information for water quality management in mega-water-transfer projects, directly supporting the Jiaodong Main Line and Yellow River Crossing operations.

## 1. Introduction

Heavy metals (HMs) exhibit complex environmental behaviors due to their strong adsorbability, fluidity, and speciation-dependent bioavailability. In lacustrine ecosystems, due to the poor flow of water, the relatively low oxygen content is more susceptible to pollution, HMs in the water in the form of ions are absorbed and eventually settle into sediments, the concentration of HMs in the sediment is often three to five orders of magnitude higher than that in the overlying water [1], there are various distributions of HMs, and the sediment is often the final destination of HMs [2,3,4]. Sediments thus serve as both sinks and potential secondary sources—environmental perturbations (e.g., pH/redox shifts) can trigger HM remobilization, enabling trophic transfer that threatens human health via carcinogenic (As, Cr) and neurotoxic (Pb, Cd) effects [5,6,7].

Dongping Lake’s dual role as the terminal regulating reservoir of the South-to-North Water Diversion Project’s Eastern Route and a key Yellow River irrigation hub makes it critically vulnerable. The water quantity of Dongping Lake changes greatly in any given year. Due to the influence of the South-to-North Water Diversion Project, the changes in hydrological and hydrodynamic conditions have accelerated the frequent interaction between rivers and lakes, as well as wetland plants and lakes. This complicates the migration and transformation mechanism of heavy metal (HM) pollution within the lake ecosystem [3,8,9].

With the rapid economic development of the area around Dongping Lake in recent years, the aquaculture, poultry farming, agricultural runoff, and industrial discharge are increasing, and the water environment problems have produced a serious rebound effect, especially the pollution input of Dawen River [10,11]. Monitoring the concentration of trace metals in the sediments of Dongping Lake and assessing their pollution status are urgent issues to address to ensure the maintenance of the lake ecosystem function in the future. Researchers [12,13] investigated the concentration of trace metals in 59 surface sediment samples from different areas of Dongping Lake, indicating that there are moderate ecological risks of HM pollution arising in the sediments of Dongping Lake. Although recent sediment studies indicated that cadmium is the primary ecological risk factor, contributing significantly to the Risk Index (RI) at 67% [14], the following critical knowledge gaps persist:

- Lack of chemical speciation data concerning HM mobility and toxicity.
- Insufficient research on the release risk and transport of heavy metals from sediments.
- Outdated health risk models ignoring exposure pathway interactions.

This study addresses these gaps through an integrated assessment of Dongping Lake’s HM pollution. Our objectives are as follows:

1. Characterize the spatial heterogeneity of 12 heavy metals (Ti, V, Cr, Mn, Fe, Co, Ni, Cu, Zn, As, Cd, Pb) in water–sediment systems and conduct a pollution assessment.
2. Determine the chemical speciation (BCR sequential extraction) to evaluate the remobilization risks.
3. Model the multi-pathway health risks (ingestion/dermal/inhalation) for residents during high-exposure periods.
4. Predict the pollution sources through correlation analysis and existing studies.

## 2. Material and Methods

### 2.1. Study Area

**Figure 1:**
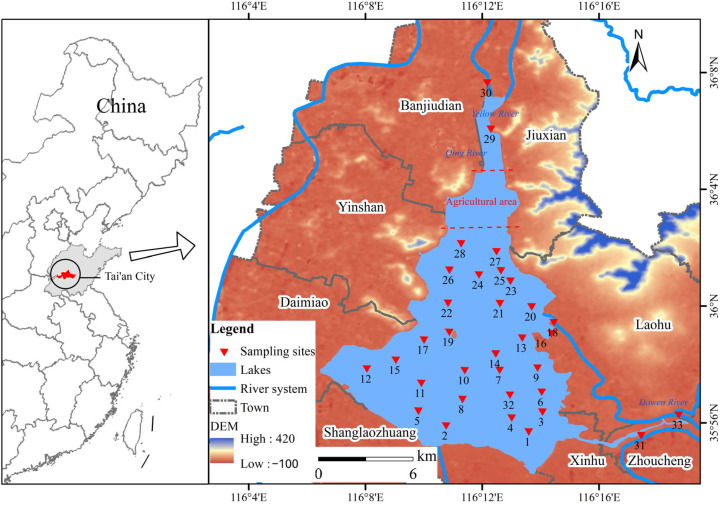
Study area and distribution map of the sampling points. (Red dashed line: aquaculture zone boundary).

Dongping Lake (35°30′~36°20′ N, 116°00′~116°30′ E), the second-largest freshwater lake in Shandong Province and the only natural lake in the lower Yellow River basin, serves as the terminal regulating reservoir for the Eastern Route of China’s South-to-North Water Diversion Project (Figure 1). Located in Dongping County, Tai’an City, this 124 km^2^ lake provides critical ecosystem services, including water supply for >10 million people and regional tourism. Hydrological connectivity shapes its pollution dynamics. Dongping Lake is connected with Xiaoqing River to the Yellow River in the north, and with Dawen River in the east, which is the only runoff into the lake except for various spillways and the diversion of water from the middle route of the South-to-North Water Diversion Project. Morphometric features influence the contaminant distribution, with post-dredging depth heterogeneity, with the depths exceeding 5.0 m in dredged zones contrasting with a mean depth of 2–4 m in undredged natural areas. Anthropogenic pressures have intensified since 2010: industrial discharges (metal processing, chemical plants), agricultural runoff (fertilizers/pesticides from farmland), and aquaculture expansion, resulting in documented heavy metal enrichment in sediments.

### 2.2. Sample Collection and Measurements

Sampling was conducted based on the principle of uniform grid points. Sampling design: Surface water (*n* = 33) and sediment (*n* = 31) samples were collected from 33 systematic grid sites in April 2024, excluding artificially fenced aquaculture zones (36°30′–37°00′ N) to prevent ecological disturbance, and unconsolidated substrates along the Qinghe/Dabeng Rivers where stable sediment retrieval was infeasible, so only water samples were collected for analysis. Water samples were obtained 20–40 cm below the surface using a 1 L water sampler (600 mL/site), filtered through 0.45 μm cellulose acetate membranes, and acidified to pH < 2 with trace-metal-grade HNO_3_. Sediments were collected with a grab-bucket sampler (0–15 cm depth), with the oxidative surface layers (top 1–3 cm) removed prior to storing the anaerobic cores in Ziplock^®^ bags (San Diego, CA, USA). All the samples were kept and transported at 4 °C at low temperature. After the samples were brought back to the laboratory, the sediment samples were freeze-dried, and then the dried sediment samples were first extracted with forceps, removing large stone particles, shells and plant residues, and then ground in an agate bowl and passed through a 200-mesh nylon screen for later analysis.

The sediment samples were processed by the microwave digestion method with an HNO_3_-HF-HCl system, and the chemical speciation was studied by the BCR method. For the detailed experimental procedures, see Text S1 and Table S1 (Supplementary Materials).

### 2.3. Data Analysis

#### 2.3.1. Ecological and Health Risk Assessment

##### Assessment of Surface Water Pollution Levels

The Heavy Metal Pollution Index (*HPI*) is a widely adopted metric for water quality assessment [15]. This method comprehensively evaluates the influence of water quality pollution caused by various HMs in the water body based on the weighted mean value.

Based on the evaluation criteria set by the *HPI* for Dongping Lake’s surface water, ten HMs (Ti, V, Cr, Mn, Fe, Co, Ni, Cu, Zn, and As) were prioritized for analysis due to their high detection rate and good data distribution within the study area for the evaluation of the surface water HM pollution.

The *HPI* method is usually calculated using the following three steps.

Calculate the weight of the *i*th HM index.

(1) $$W_{i}=\frac{k}{S_{i}}$$

Calculate the quality grade index of the *i*th HM index.

(2) $$Q_{i}=100\left(\frac{\rho_{i}}{S_{i}}\right)$$

Weighted calculation of the *HPI*.

(3) $$HPI=\frac{\sum_{i=1}^{n}\left(Q_{i}W_{i}\right)}{\sum_{i=1}^{n}W_{i}}$$

where $S_{i}\;$is the maximum allowable mass concentration (μg/L) in water bodies. In this paper, the GB 3838-2002 [16] “Surface Water Environmental Quality Standard” Class III standard limit is used; *k* is the proportion of Changshu determined by the conditions, which is usually 1 for simple calculation; $\rho_{i}\;$is the monitoring mass concentration (μg/L) of HMs in the water; and *n* is the number of evaluation indicators. Usually, the pollution critical index *HPI_c_* = 100, but when the *HPI* > 100, it is considered that the degree of HM pollution in the water body has exceeded its highest acceptable level.

#### 2.3.2. Sediment Ecological Risk Assessment

##### Potential Ecological Risk Index

The selection of geochemical background values is critical for accurate heavy metal pollution assessment. Given Dongping Lake’s position within the Yellow River floodplain and its sedimentary composition dominated by fluvial deposits from the middle–lower Yellow River [17], we adopted the regional sediment background values of the Yellow River [18] as reference baselines for the surface sediment HM evaluation.

The Potential Ecological Risk Index (PERI), pioneered by Hakanson [19] (1980), quantifies synergistic ecological threats from multi-metal interactions through integrated toxicological and environmental exposure assessment. The calculations are as follows:

$$R_{I}=\sum E_{r}^{i}$$

$$E_{r}^{i}=T_{r}^{i}C_{f}^{i}$$

$$C_{f}^{i}=C_{0}^{i}/C_{n}^{i}$$

where $C_{f}^{i}$ is the pollution coefficient of HMs (mg/kg); $C_{0}^{i}$ is the actual measured content of the element (mg/kg); and $C_{n}^{i}$ is the evaluation reference ratio of this element. In this study, the background value of the sediments in the lower Henan reach of the Yellow River Basin is taken as the reference. $E_{r}^{i}\;$is the potential harm coefficient of the element. $T_{r}^{i}$ is the reference for the toxicity response coefficient of this element [20]. $R_{I}$ is the potential ecological hazard index of this element. The background value and toxicity coefficient are listed in Table S2.

##### Geo-Accumulation Index

The geo-accumulation index method was proposed by Muller [21]. It uses the ratio relationship between the HM content in sediments and the background value of sediments to express the method of evaluation of the degree of HM pollution as follows:

$$I_{geo}={log}_{2}\left[\frac{C_{n}^{i}}{K\times B_{n}^{i}}\right]$$

where *I_geo_* is the geo-accumulation index of HM *i*,${\;C}_{n}^{i}$ is the measured value of HM *i* in soil (mg/kg),$\;B_{n}^{i}$ is the background value of HM *i* (mg/kg), and *K* is the coefficient utilized to eliminate the influence of rock differences in different places, which is generally 1.5. In this study, the background value of Yellow River sediments is used as the reference background value for the accumulative study of surface sediments in Dongping Lake. The evaluation index of the sediment’s potential to cause ecological harm is listed in Table S3.

##### Risk Assessment Code

The risk assessment coding (RAC) method adopted the proportion of exchangeable HMs to the total amount to assess the risk of environmental harm, which has been proved to be a good index with which to assess the mobility and potential release risk of HMs. The higher the RAC value, the stronger the migration and biological activity of HMs, and the greater the risk value, and vice versa. The calculation is as follows:

𝑅𝐴𝐶 = [𝐶_𝐹1_/(𝐶_𝐹1_ + 𝐶_𝐹2_ + 𝐶_𝐹3_ + 𝐶_𝐹4_)] × 100%

where *C_F_*_1_, *C_F_*_2_, *C_F_*_3_, and *C_F_*_4_ are the contents of the weak acid fraction, reducible fraction, oxidizable fraction, and residual fraction, respectively. Five levels of risk based on the proportion of the weak acid fraction (*F*1) are used. The evaluation index of pollution by the chemical form of heavy metals in sediments is listed in Table S4.

#### 2.3.3. Monte Carlo Health Risk Assessment

The probabilistic health risks from drinking water exposure were quantified using the U.S. EPA-recommended methodology (EPA/600/R-22/236), with Monte Carlo simulation addressing the parameter uncertainties. The methodological justification and parameter validation are detailed in Supplementary Text S2.

The total human health risk index (*R*) is composed of the carcinogenic risk (*R^C^*) and non-carcinogenic risk (*R^n^*), and the *ADD_i_* represents the average daily intake of metals through drinking water, calculated by the following formula:

$$R_{i}^{c}={ADD}_{i}\times {SF}_{i}$$

$$R_{i}^{n}=\frac{{ADD}_{i}}{{RfD}_{I}}\times 10^{-6}$$

$${ADD}_{i}=\frac{C_{i}\times IR\times EF\times ED}{BW\times AT}$$

where $R_{i}^{c}$ and $R_{i}^{n}$ are the total health risk of chemical carcinogenic and non-carcinogenic element *i* produced by the drinking water pathway, respectively, ${ADD}_{i}$ is the mean daily intake through drinking water [mg·(kg·day)] and $C_{i}$ represents the content of element *i* in sediments (mg/L). *IR* represents the sediment particle intake (mg/d); *EF* is the exposure frequency (days/year); *ED* is the duration of sediment exposure (year); *BW* is the average body weight (kg); *AT* is the average exposure time (days); ${RfD}_{I}$ represents the intake reference dose of metal *i* [mg/(kg·day)]; and ${SF}_{i}$ represents the intake slope coefficient of metal *i* [mg·(kg·day)].

As there are various types and ways of exposure to HMs in the human living environment, the total exposure hazard index (*TEHI*) can be defined by the sum of the $R_{i}^{n}$ of each exposure path of all the metals studied, with *TEHI* < 1 representing a negligible risk to human health or an acceptably low risk. However, when *TEHI* > 1, it may have adverse effects on human health, as shown in the following equation:

$$TEHI=\sum R_{i}^{n}=\sum_{i=1}^{N}\frac{{ADD}_{i}}{{RfD}_{i}}$$

For carcinogenic metals (Pb, Cr, and Pb), the risk of cancer is estimated as the individual’s lifetime probability of developing cancer from potential exposure to carcinogens. The total carcinogenic risk index (*TCRI*) is calculated thus:

$$TCRI=\sum_{i=1}^{N}\left({ADD}_{i}\times {SF}_{i}\right)$$

When 10^−6^ < *TCRI* < 10^−4^, the risk is within the acceptable range, and when the TCRI value is larger than 10^−4^, it indicates that the human tolerance for the carcinogenic risk is exceeded. This study was based on the data of the U.S. EPA IRIS database, and the toxicological parameters of HMs in relevant studies were collected as supplements. For specific considerations of the model, see Text S2; for details pertaining to the values and distributions of various parameters, see Table S5 (Supplementary Materials).

Monte Carlo simulations (10,000 iterations) were implemented via Oracle Crystal Ball^®^ integrated with MS Excel^®^ (Build 16.0.4266.1001) to quantify the parameter uncertainties in the health risk assessment. The 95% confidence intervals of the probabilistic outputs were stabilized through convergence testing. The Monte Carlo method was used to conduct uncertainty analysis and sensitivity analysis of each parameter for the human health risk assessment of Dongping Lake. A positive sensitivity indicates that the factor is positively correlated with the risk result, and the greater the absolute value of the sensitivity, the higher the contribution rate to the risk result.

## 3. Results and Discussion

### 3.1. Distribution and Contamination of HMs

The concentrations of 12 heavy metals in Dongping Lake followed this hierarchy: Fe ([mean] μg/L) > Zn > As > V > Ti > Mn > Ni > Cu > Cr > Co > Pb > Cd (Table 1). All the metals fell below regulatory limits (China GB 3838-2002/WHO drinking water standards), confirming negligible health risks. To evaluate the water pollution level of Dongping Lake and determine the correlation between different water pollutants and HMs, this study determined the following surface water quality parameters: total dissolved solids (TDSs), electrical conductivity (EC), dissolved oxygen (DO), pH and water temperature. The HPI averaged 7.61 (range: 3.67–19.04; Table S6), with 85% of sampling sites scoring <10, indicating consistently low pollution levels and a homogeneous spatial distribution across the lake.

**Figure 2:**
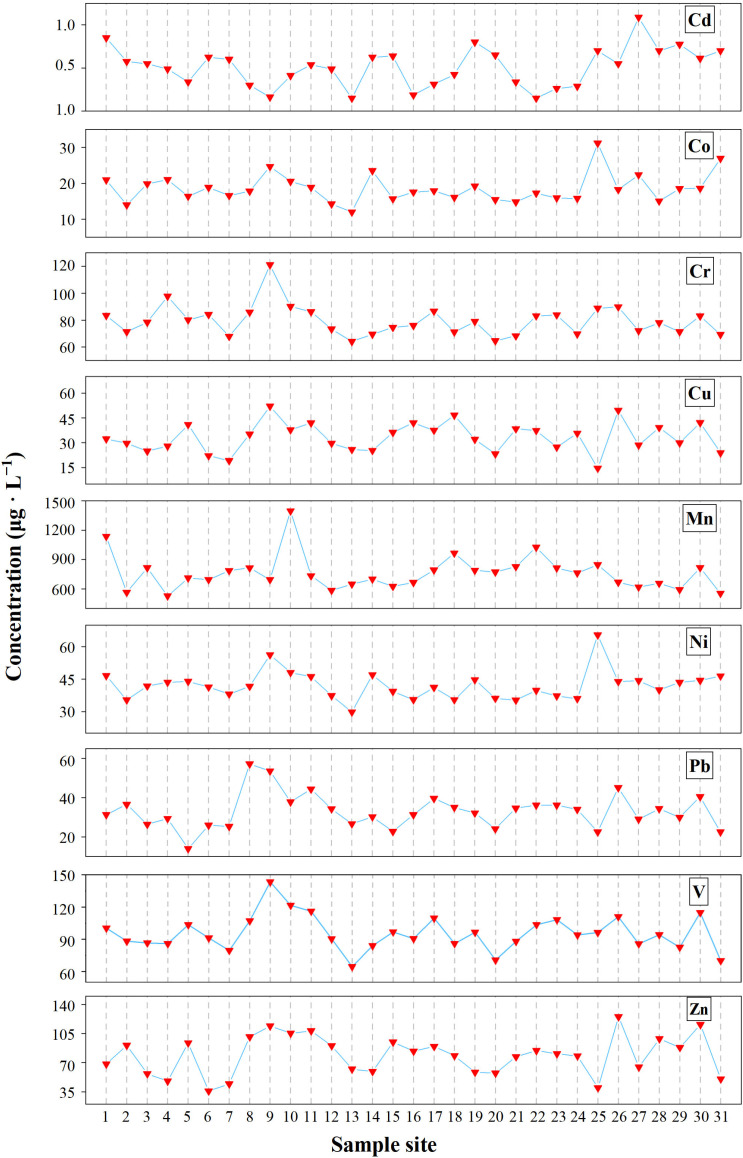
Total concentration and trend of HMs in sediments.

The measurement results and descriptive statistical results of the HM concentrations in the sediments of Dongping Lake are shown in Figure 2 and Table 1, from which it can be seen that the average concentrations of all the HMs are as follows: Mn > Cr > V > Zn > Ni > Cu > Pb > Co > Cd are 1.33, 1.3, 1.28, 1.34, 1.5, 1.58, 1.65, 1.67, and 4.53 times the background value of the Yellow River sediments, respectively. The content of Cd is significantly higher than the background concentration, indicating that there is a certain HM enrichment in the surface sediments of Dongping Lake. According to the coefficient of variation, the contents of HMs at different sampling sites are small, especially those of Co, Cu, Zn, and Pb, whose coefficients of variation are 30, 27, 27, and 22, respectively, belonging to the medium variation. It is stable in the spatial distribution and is not easily affected by external input.

Compared with other lakes in China and lakes along the South-to-North Water Diversion Project, it can be concluded that water transferred from Yangzhou, Jiangsu Province, successively passes through Hongze Lake, Luoma Lake, Nansi Lake, and Dongping Lake, and the HM pollution degree in the sediments generally decreases gradually, indicating that the operation of the Eastern Route of the South-to-North Water Diversion Project has reduced the HM content in the sediments of each storage reservoir. Due to the different shapes of the lake body and the difference in hydrodynamic conditions, HM pollution is the most serious in Nansi Lake. However, compared with Taihu Lake and Poyang Lake, the level of pollution is similar. Compared with other lakes, the environmental condition of Dongping Lake is better.

**Figure 3:**
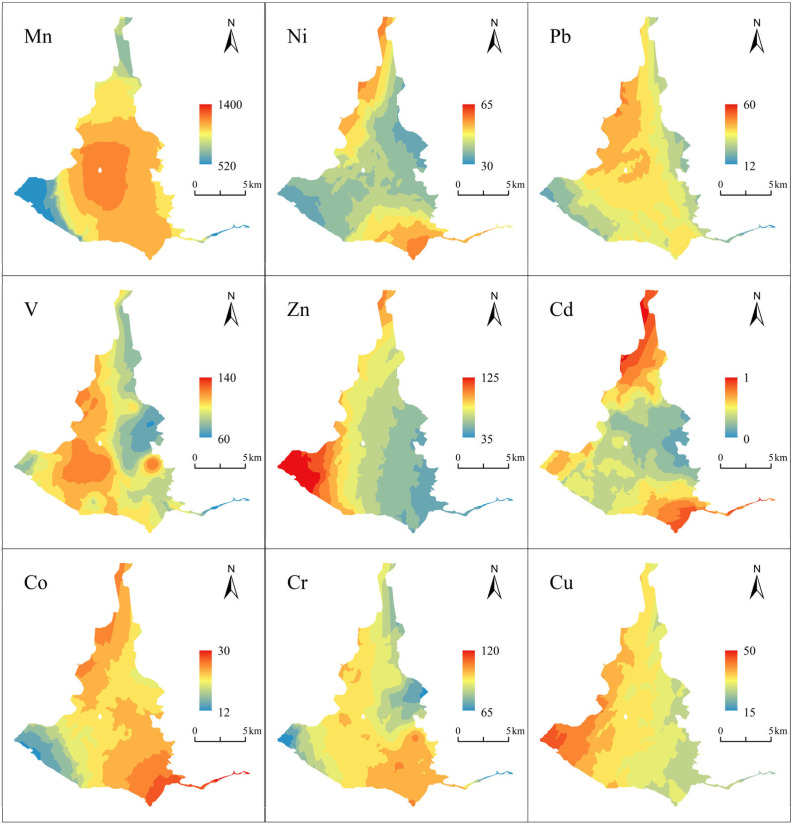
Spatial distribution of HM concentrations in surface sediments of Dongping Lake.

Figure 3 shows the spatial distributions of V, Cr, Mn, Co, Ni, Cu, Zn, Cd, and Pb in the surface sediments of Dongping Lake in this experiment. Compared with the background value of sediments from the Yellow River, it is found that Cd is 4.53 times the background value, and the pollution is relatively serious. In the southwest, the pollution level is lower than in the two concentrated areas but still higher than that in most other areas of the lake. The spatial distribution of Zn shows a trend of increasing gradually from east to west, and there is an area of enrichment in the southwest side. The area with higher V content is the western part of the middle of the lake, and the pollution in a plane distribution gradually aggravates from the northeast to southwest side. The area with high Pb content is located in the northwestern part of the lake, and it gradually declines to the southeast and both sides of the lake. The spatial distribution of Cu is similar to that of Zn, and it gradually increases from east to west. The spatial distribution characteristics of Co and Ni are similar, with high content in the northwest and southeast and low content in the southwest and northeast. The content distribution of Mn decreases gradually from the center of the lake to the surrounding area, and the spatial distribution of Cr is uneven, and the pollution distribution is mostly spot-like, but the most heavily polluted area remains in the southeast, near the inlet of the Dawen River. The spatial distributions of Mn and V are similar, the distributions of Zn and Cu are similar, and the distributions of Cd, Co, Cr, Ni, and Pb are similar.

According to the comparison between the nine HMs in the surface sediments of Dongping Lake and the background values, the pollution levels of V, Cr, Mn, Co, Ni, Cu, Zn, Cd, and Pb in the surface sediments of Dongping Lake are at a light level of pollution, and the pollution levels of Cd are heavy, among which 64.5% of all the points of Cd are rated as “seriously polluted”. In particular, in the northern part of Dongping Lake and near the entrance of the Dawen River, the Cd content is many times more than that in other areas, which suggests that there is point-source pollution of Cd.

Further, to explore the bioavailability and biotoxicity effects of HMs in sediments, the results of this study were compared with the revised sediment quality guidelines proposed by Yang Jinxi for seven major river systems in China [27]. The critical effect concentration and possible effect concentration are shown in Table S7. The TEL is the boundary line of the pollutant concentration to determine “with” or “without” obvious effect. The PEL is the threshold at which the hazard is considered to occur “frequently”. The HM contents in this study are all lower than the PEL, that is, the adverse effects of HMs in sediments on aquatic organisms do not occur “frequently”. However, 94% and 51.61% of the Ni and Zn contents are significantly higher than the TEL, indicating that Ni and Zn in sediments have potential adverse biotoxic effects and may produce negative biological effects. It is noteworthy that although the single factor risk index of Ni and Zn is relatively small and the contribution rate to the potential ecological risk is small, it still causes significant harm to aquatic organisms. Therefore, the study of different forms of HMs should be strengthened further to understand the bioavailability and environmental toxicity of HMs.

**Figure 4:**
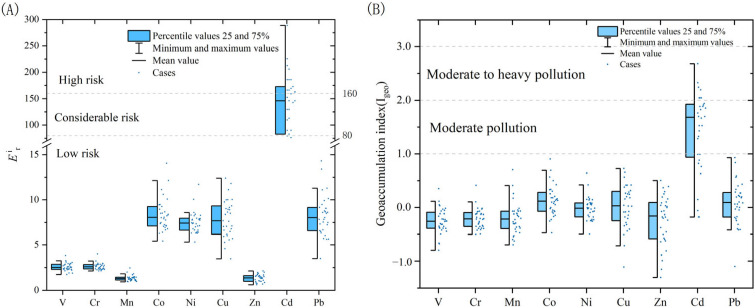
(**A**) The evaluation results of the sediment potential ecological hazards. (**B**) The calculation results of the geo-accumulation index method.

The evaluation results of the sediment potential ecological hazards are shown in Figure 4A. According to the calculation results of the potential ecological hazard index method, the potential hazard coefficients of HM elements in the surface sediments of Dongping Lake are as follows: Cd (135.95) > Co (8.37) > Pb (8.25) > Cu (7.9) > Ni (7.51) > Cr (2.62) > V (2.55) > Zn (1.34) > Mn (1.33), as determined by comparing with the rating of the potential ecological hazard index method. All the other HMs except Cd are at a low risk level, Cd is at a high risk level, and the average value of the potential ecological hazard index is 175.82, suggesting a medium risk level.

The evaluation results of the geo-accumulation index method are shown in Figure 4B. According to the calculation results of the geo-accumulation index method, the geo-accumulation indexes of HM elements in the surface sediments of Dongping Lake are as follows: Cd (1.4) > Co (0.11) > Pb (0.101) > Cu (0.03) > Ni (0) > Mn (0.19) > Cr (0.203) > Zn (0.22) > V (0.24). In addition to the enrichment of the Cd pollution levels being at a moderate level, Co, Cu and Pb are mildly enriched, and the other HMs are below mildly enriched, indicating that V, Cr, Mn, and Zn pose potential risks.

The potential ecological hazards of V, Cr, Mn, and Zn show that the *E_r_* values of these four elements are all less than 5, which generally do not cause ecological hazards. Therefore, it is inferred that these elements may mainly come from rock minerals in nature. Co, Ni, Cu, and Pb are known as anthropogenic factors, and their *E_r_* values are all in the range of 5–10 and show enrichment levels in sediments, while the *E_r_* values of Cd are in the range of 80–160, indicating enrichment and greater potential risks. At the same time, these elements have a similar spatial distribution, where the maximum element content is found in the southeast of the lake, and decreases to the southwest and northeast, while it increases in the north.

One of the reasons for this distribution trend is that the southeastern part of the lake is the entrance to the mouth of the Dawen River, a tributary of Dongping Lake. The Dawen River is the only tributary of Dongping Lake. The tributary flows through the industrial areas and densely populated areas of many surrounding counties and becomes a sewage discharge channel for domestic sewage and wastewater from industrial and mining enterprises. The discharge of industrial wastewater, domestic wastewater and agricultural sewage accounts for 34%, 44%, and 22% of the total, and 80% of the reaches of the Dawen River are severely polluted. The Dawen River basin is distributed with various coal mining, paper making, chemical, and steel industries, etc., which contribute greatly to the Cu, Zn, and Pb pollution. Meanwhile, the southeast is close to the main urban industrial zone, Dongping power plant, a machinery factory, and various processing factories; the wastewater generated by these factories enters Dongping Lake directly or indirectly through surface runoff and atmospheric settlement, resulting in more severe pollution in the southeastern area of the lake. The surface runoff of the Dawen River carries a large amount of HM pollutants and organic pollutants. When the river enters Dongping Lake, due to the narrowing of the river channel and the slow flow rate, HM pollutants and other pollutants are first deposited in the southeastern part of the lake. However, during the study period, the increments of incoming water and the flow rate will cause greater disturbance to the sediments, which will increase the northwest diffusion range and speed of HMs. The migration behavior of HMs from the Dawen River to Dongping Lake can well explain the trend in the spatial distribution of the metals wherein the concentrations decrease from the southeast of the lake to the west and north.

### 3.2. Chemical Speciation of HMs in Surface Sediments

HMs threaten aquatic ecosystems and human health. They cause toxic effects such as neurotoxicity, growth inhibition, and reproductive disorders in aquatic organisms. Through bioaccumulation, they enter the food chain and may eventually harm humans via consumption of contaminated water or seafood, leading to chronic health issues, including organ damage and increased cancer risk [28]. Therefore, quantitatively assessing the environmental risks posed by these pollutants is of crucial importance for ensuring ecological security and public health.

**Figure 5:**
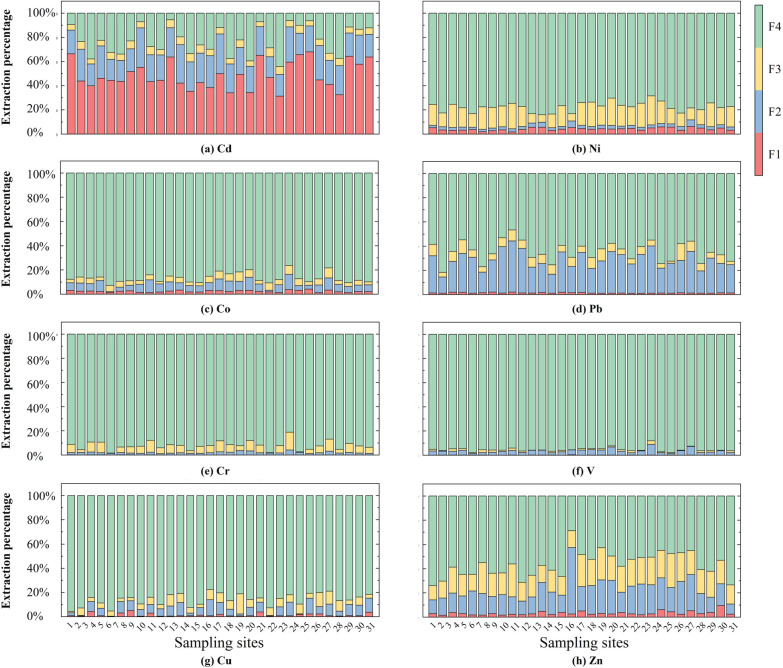
Distributions of HMs in surface sediments of Dongping Lake. (**a**) Distributions of Cd in surface sediments of Dongping Lake; (**b**) Distributions of Ni in surface sediments of Dongping Lake; (**c**) Distributions of Co in surface sediments of Dongping Lake; (**d**) Distributions of Pb in surface sediments of Dongping Lake; (**e**) Distributions of Cr in surface sediments of Dongping Lake; (**f**) Distributions of V in surface sediments of Dongping Lake; (**g**) Distributions of Cu in surface sediments of Dongping Lake; (**h**) Distributions of Zn in surface sediments of Dongping Lake.

The total contents and distributions of eight HMs in 31 surface sediment samples from Dongping Lake are shown in Figure 5. Among them, the components in the weakly acidic state (F1), the reducible state (F2), and the oxidizable state (F3) can be extracted and can also be called the available state, which poses certain potential risks to the ecological environment. From the weak acid state to the oxidizable state, the migration and bioavailability of different forms of occurrence are gradually enhanced, and the weakly acidic state is the most sensitive to changes in the external environment. Residual (F4) HMs are stable in form, difficult to migrate and transform under normal conditions, and have the lowest bioavailability [12].

Except for Cd, residual fractions (F4) dominated most HMs (V: 95.42%, Cr: 92.21%, Co: 86.71%, Ni: 77.55%, Cu: 85.76%), indicating geogenic origins and low bioavailability. Cd exhibited high environmental risk, with a 48.6% acid-soluble fraction (F1), meaning it is easy to be directly absorbed by plants and causes the greatest potential harm to the environment [29]. Pb primarily existed as a reducible fraction (27.55%). The reducible ferric manganese hydroxide in the soil has a strong adsorption capacity for lead ions, so that Pb is mostly present in the combined state of ferric and manganese oxides [30,31]. Another possibility is that the pH of Dongping Lake water is weakly alkaline, the average pH is 7.99, and there are more calcium carbonate ions therein, which promotes the complexation between Pb and solid surface ions. Both of these may lead to a high proportion of Pb in the reducible state. A similar phenomenon in terms of Pb enrichment in the reducible fraction, attributed to the comparable hydrochemical conditions, has also been reported in alkaline water bodies such as the Pearl River Delta [32].

The morphological distributions of HMs in sediments at different sampling points shows that Cd is mainly in the extractable and reducible states of weak acids, and the element properties are unstable and readily migrate, which is attributed to the inherent elemental properties of Cd and also reflects the contribution of surrounding human activities. Pb is mainly reducible. As V, Cr, Co, Ni, and Cu are primarily found in the residual state, their distribution is uniform, and their potential to threaten the environment is consequently low. The residual states of Zn and Pb account for 56.74% and 64.44%, respectively, which do not exceed 75%, indicating that their chemical activity is higher than that of the top-five HMs. Attention should be paid to Cd inputs, such as agricultural inputs through stormwater runoff or industrial pollution through direct current, as well as the potential pollution by Pb and Zn.

The evaluation results are shown in Supplementary Materials Table S8. The RAC average value of Cd is much higher than that of the other elements, in which Co, Ni, Cu, Zn and Pb have low risk levels, while V and Cr are in a risk-free state. The average RAC of Cd is 48.64%, the highest is 67.92%, 11 sites are at moderate/intermediate risk, and the rest are at high risk.

The bioavailability of V/Cr is extremely low (negligible risk), there is slight pollution from Co/Ni/Cu/Zn/Pb, and there is severe point-source Cd pollution with high spatial heterogeneity, fully demonstrating that the form of HMs is an important factor causing ecological risks. Cd hotspots occur near southeastern/northern farmlands and villages, likely induced by agricultural runoff (fertilizers, pesticides, manure) altering the HM speciation and mobility.

#### 3.2.1. Human Health Risk Assessment of HMs in Sediments

**Figure 6:**
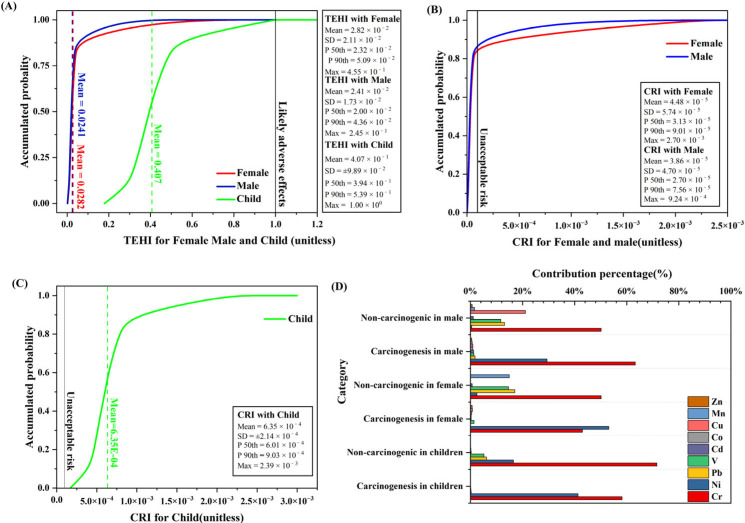
(**A**,**B**) Cumulative frequency maps of non-carcinogenic risks for adult men, women and children; (**C**) cumulative frequency map of cancer risk in children; and (**D**) sensitivity analysis chart.

The health risks from sediment heavy metals are quantified in Figure 6A–C and Table S9. The non-carcinogenic hazard index (HI) of nine HMs is Cr > Mn > V > Pb > Ni > Co > Cu > Cd > Zn, with significant population susceptibility differences: children (mean HI = 0.408) > adult females (0.0284) > adult males (0.024). Additionally, the maximum value is below the critical threshold (HI = 1.0). This confirms the negligible non-carcinogenic risks from Dongping Lake sediments across all demographics.

According to the results of the carcinogenic health risk assessment of HMs in the surface sediments of Dongping Lake, the average carcinogenic risk index (CRI) of the three carcinogenic HMs is as follows: Cr > Ni > Cd, and the carcinogenic risk types of the three groups of people show that the CRI of children is significantly larger than that of adults, while for adult women it is slightly larger than that for adult men. This is due to children’s physiology and behaviors, such as playing in lakes and inadvertent water ingestion. Despite the population-specific TCRs exceeding the threshold (10^−4^)—children (6.33 × 10^−4^) > women (4.47 × 10^−4^) > men (3.86 × 10^−4^)—probabilistic analysis shows a 0% unacceptable risk probability for children versus 6–7% for adults. These findings indicate that sediment-borne metals pose quantifiable carcinogenic threats, demanding targeted intervention for child populations.

Children exhibit significantly higher susceptibility to both carcinogenic and non-carcinogenic risks than adults, while adult males show lower vulnerability than females. The difference between adult men and women in the parameters is mainly due to body weight, which is also confirmed by other studies. Groups with lower body weight face higher levels of health risks from pollutants [33].

#### 3.2.2. Sensitivity Analysis

Sensitivity analysis quantified the parameters’ influence on the risk outcomes (Figure 6D). Among the non-carcinogenic health risks, Cr has the highest sensitivity, followed by Mn and Pb, which together account for over 90% of the risk. The most sensitive values of the carcinogenic health risk are Cr and Ni, which account for more than 92% and are the primary factors influencing carcinogenic health risks.

Children exhibit significantly higher carcinogenic and non-carcinogenic risks than adults due to their prolonged exposure windows and physiological susceptibility. Priority protection is warranted for this high-risk group. Particularly, Cr and Cd dominate the health threats, and the carcinogenic and non-carcinogenic risks due to Cr need to be paid special attention. Although dermal exposure poses minimal risks, occupational groups (fishers, tourism workers) and populations near Cd-emitting industries (e-waste processing, non-ferrous smelting) require paying more attention to protection.

### 3.3. Correlation Analysis and Pollution Source Identification

**Figure 7:**
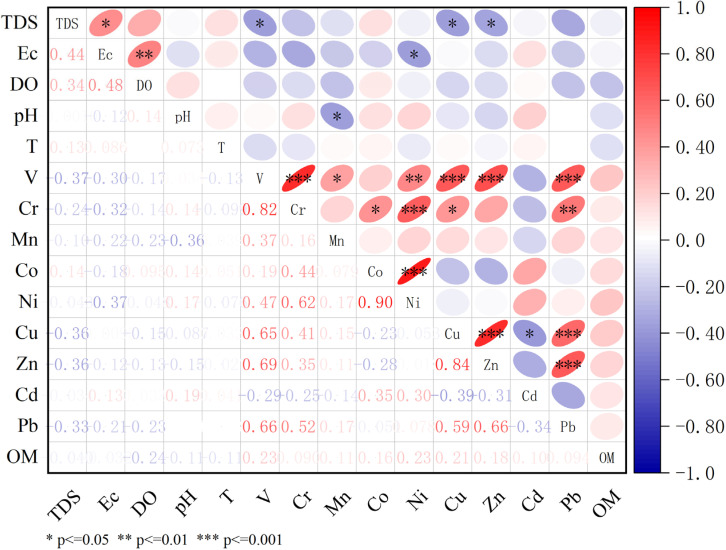
Heatmap displaying the correlations among HMs and various parameters.

The correlation heatmap between basic environmental parameters and HMs (Figure 7) was analyzed to elucidate their controlling factors and common geochemical behaviors. Figure 7 shows that there is a correlation between the TDS and EC in the water quality parameters measured on site. The solid content of water and the amounts of salts in water will determine the conductivity of water, so the two are positively correlated. The significant positive correlation between the EC and DO indicates that the EC is also higher in areas with higher DO, and the corresponding TDS is also higher, indicating that the spatial distribution of these three indicators is similar.

The correlation between different metals provides key information for studying the pollution source and determining whether there are sources that simultaneously emit one or more HMs. In this study, Pearson correlation coefficient analysis was performed for the association between nine metals in sediments and organic matter (OM) in sediments. When the correlation coefficient *R*^2^ of two metals at the double-tail level exceeds 0.7 (*p* ≤ 0.01), it indicates that the two metals are more likely to have a common source, and there is a moderate coexistence between 0.3 and 0.7. At the same time, the distribution characteristics of different HMs in the study area are discussed. The strong correlation between Co and Ni results from the pollution caused by the production of lithium batteries with ternary materials such as nickel cobalt manganese or nickel cobalt aluminate as the positive electrode of batteries. Nickel–cadmium batteries can also explain the weak correlation between them.

The average levels of Cd, Ni, Cu, and Co in sediments match those in water due to their natural storage differences. However, Mn, Pb, Zn, and other elements show different patterns. This discrepancy arises from the following. (1) Long-term variations in the HM inputs into the lake, where most HMs attach to suspended particles and settle due to hydrodynamic forces and gravity. Sediments, as stable environmental media, reflect the long-term HM accumulation, while the water HM levels indicate only the sampling period’s HM import status. (2) Different HMs distribute differently in water and suspended particles. Pb and Ni, with the strong adsorption capacities, accumulate in particles over time. (3) The South-to-North Water Diversion Project affects the HM content and distribution in the lake during the sampling period, causing variations in the average element levels.

Cd and Pb often coexist in primary mineral deposits, with Cd being highly toxic and carcinogenic. Ecological hazard assessments indicate that Cd exhibits severe ecological hazards (mean *E_r_* = 135). Therefore, it is certain that Cd is greatly affected by human factors. Previous studies found [34,35] that Cd is related to the use of phosphate fertilizer and insecticides in agricultural production. The spatial heterogeneity in the Dongping Lake sediments correlates with the annual phosphate fertilizer use (127,000 tons) and pesticide application, driving Cd enrichment via runoff. Industrial contributions (electroplating, battery production) further elevate the bioavailable Cd fractions. The spatial distribution of Cd in the sediments of Dongping Lake correlates strongly with the agricultural and sewage discharge areas in its northern and southern regions, indicating anthropogenic activities to be the main cause of severe non-point-source pollution. Although source apportionment remains debated, studies consistently highlight high-heavy-metal pesticides and phosphate fertilizers as major contributors to Cd accumulation in surface waters [36]. Industrial and domestic wastewater discharges also significantly contribute, especially in rapidly urbanizing basins in China, resulting in mixed point and non-point pollution patterns [3,37]. Meanwhile, some studies have shown that the complexation of Cd and DOM in sediments is the main cause of the sudden outbreak of Cd pollution. The complexation of OM and Cd in sediments will further release Cd [38,39,40], resulting in significant cadmium pollution. The OM value (4.46–11.88%) in this study is much higher than the background value of the soil OM in Shandong Province (1.16%) [41], and the lake-bottom environment is conducive to complexation, suggesting that OM–Cd complexation occurs in the sediments.

## 4. Conclusions

This study comprehensively assessed the heavy metal (HM) pollution across the water–sediment continuum in Dongping Lake through multi-media. Additionally, their pollution status, morphological characteristics, risk assessment and source analysis were studied. A variety of methods were used for coupling analysis of the pollution status in this area, and the following conclusions were drawn. (1) The average contents of 12 HMs in Dongping Lake water are lower than the standard limit of drinking water quality recommended by the WHO, and the water quality is relatively clean. Cd is the main pollutant, and the potential ecological risk is at the medium risk level. The geo-accumulation index shows that Cd is moderately enriched, while Co, Pb, and Cu are mildly enriched. The results of the sediment quality guidelines indicate that Ni and Zn in sediments have potential adverse biotoxic effects. (2) The analysis of the HM forms present in sediments shows that, according to the ecological risk assessment results of the chemical forms of HMs in surface sediments, V and Cr tend not to pollute Dongping Lake; Co, Ni, Cu, Zn, and Pb only cause slight pollution; and Cd generates severe point-source pollution. (3) The results of the health risk assessment show that the intake of metals in the surface sediments of Dongping Lake can cause cancer in the local population, especially among children. However, the pathways for human exposure to sediments are limited. Thus, the overall carcinogenic risk remains low; the non-carcinogenic risk is negligible; and the health risk mainly comes from Cr and Cd, which warrant special attention. (4) The results of a correlation analysis show that industry contributes more to Cu, Zn, and Pb, and the high Cd pollution in the north and southeast of Dongping Lake arises from the distribution of agricultural land and the discharge of agricultural sewage. Based on the data analysis and the existing research, it is concluded that the agricultural pollution caused by the farming area around Dongping Lake needs to be controlled, so as to avoid the continuous diffusion of pollution along with the hydrodynamic conditions, which will subject the water environment to more severe pollution.

## Figures and Tables

**Table 1:** Statistics concerning the HM content in the water and surface sediments of Dongping Lake.

Water	Cr	Mn	Fe	Co	Ni	Ti	V	Cu	Zn	As	Cd	Pb
$\bar{x}$*_i_*^a^ (μg/L)	0.464	1.332	15.002	0.097	1.145	1.335	1.505	0.900	3.620	3.483	0.022	0.050
*x*_max_ (μg/L)	1.390	5.860	36.810	0.180	1.540	4.300	1.890	1.510	10.260	5.530	0.040	0.120
*x*_min_ (μg/L)	0.170	0.350	7.510	0.070	0.930	0.070	0.950	0.630	0.560	2.050	0	0
SD ^b^	0.369	1.088	7.354	0.020	0.106	1.185	0.195	0.150	2.432	0.939	0.110	0.038
CV (%)	0.795	0.817	0.490	0.206	0.093	0.888	0.130	0.167	0.672	0.270	0.150	0.760
GB ^c^ (μg/L)	5	100	300	1000	20	100	50	1000	1000	50	5	50
$\bar{x}$*_i_*_＿mean＿12_ ^d^ (2020) (μg/L)	31.880	58.060	16.030		1.230			1.200	15.270	1.230	0.040	0.240
$\bar{x}$*_i_*_＿mean＿3_ ^d^ (2021) (μg/L)	38.330	4.280	111.900		14.690			3.950	35.560	1.760	0.030	0.740
$\bar{x}$*_i_*_＿mean＿9_ ^d^ (2021) (μg/L)	0.710	1.390	57.250		1.550			1.300	27.980	2.160	0.030	0.390
Sediment	Cr		Mn	Ni	Cu		Zn	V		Cd	Pb	Co
$\bar{x}$*_i_* (mg/kg)	79.44		761.46	42.07	33.19		78.84	95.65		0.512	32.99	18.57
x_max_ (mg/kg)	121.06		1396.08	29.78	52.09		125.28	143.45		1.09	57.20	31.19
x_min_ (mg/kg)	64.15		527.41	65.50	14.58		35.83	64.58		0.15	13.98	12.01
SD	11.46		180.07	6.79	8.94		23.76	16.30		0.23	9.06	4.01
CV (%)	0.14		0.24	0.16	0.27		0.30	0.17		0.15	0.27	0.22
B_i_^e^ (mg/kg)	60.65		571	28	21		59	75		0.113	20	11.1
$\bar{x}$_i＿mean＿12_ ^f^ (2020) (mg/kg)	82.10		741.07	41.08	38.5		91.17	79.69		0.25	27.32	16.47
$\bar{x}$_i＿mean＿3_ ^f^ (2021) (mg/kg)	80.67		699.97	40	37.28		68.52	79.72		0.23	26.14	16.29
$\bar{x}$_i＿mean＿9_ ^f^ (2021) (mg/kg)	79.94		692.04	37.32	34.89		68.51	73.25		0.25	25.80	14.89
Nansi Lake [22]	136.54		838.53	46.21	41.85		149.96			0.27	50.18	21.84
Taihu Lake [23]	82.10		841.60	40.60	26.10		99.50	97.30			36.60	15.40
Poyang Lake [24]	42.95				71.37		123.98			0.82	47.37	
Hongze Lake [25]	82.00			40.64	24.11		79.07			0.22	27.87	
Luoma Lake [26]	101.70			56.21	22.59					7.39	89.21	

^a^ Average concentrations of HMs in water samples measured in April 2024. ^b^ Standard deviation of the HM concentrations throughout the lake. ^c^ Environmental quality standards for surface water. ^d^ Monitoring value of HM concentration in Dongping Lake in 2021 [13]. ^e^ Background value: the mean value of elements in Yellow River sediments [18]. ^f^ Monitoring value of HM concentration in surface sediments of Dongping Lake in 2021.

## Data Availability

The original data presented in the study are included in the Supplementary Materials; further inquiries can be directed to the corresponding author.

## Supplementary Materials

The following supporting information can be downloaded at https://www.mdpi.com/article/10.3390/toxics13090762/s1. Text S1: Microwave digestion method of the HNO3-HF-HCl system [42]; Text S2: Interpretation of the applicability of the health risk assessment methods [11,43,44,45]; Table S1: European Community Reference Bureau three-stage extraction program; Table S2: Element background value and toxicity response coefficient value table; Table S3: Evaluation index of sediment potential ecological harm; Table S4: Evaluation index of pollution by the chemical form of heavy metals in sediments; Table S5: Mean, standard deviation, range and distribution of the exposure and toxicological parameters used in the human health risk assessment [46,47,48,49,50,51,52,53,54,55,56]; Table S6: Water quality parameters; Table S7: Critical effect concentration and possible effect concentration value table; Table S8: RAC value and risk level of heavy metals in soil; Table S9: Carcinogenic and non-carcinogenic risk index of heavy metals in surface sediments of Dongping Lake for children and adult men and women.
