# Supplementary material for: Assessing Multiple Risks in Regulating Reservoirs: Perspectives on Heavy Metal Contamination
Source: Toxics. 2025 Sep 8;13(9):762. doi: 10.3390/toxics13090762 (PMC12474066; doi:10.3390/toxics13090762)
Supplement: Supplementary file 1 [file toxics-13-00762-s001.zip › toxics-3819260-supplementary.pdf]

Supporting Information

Assessing Multiple Risks in Regulating Reservoirs: Perspectives on Heavy Metal Contamination

Hui Zhou [a], Zhiping Li [a,b*],AnMing Wang[a*],Jiawei Zhu [a],ZongYan Han [a] , YaLin Zhang [a] , Dongdong Chen [a]

[a] College of Geosciences and Engineering, North China University of Water Resources and Electric Power, Zhengzhou 450046, China

[b] Henan Vocational College of Water Conservancy and Environment, Zhengzhou 450046, China

* Corresponding author:

E-mail address: lizhiping@ncwu.edu.cn wam992001@163.com

Captions for Supplemental Methods and Tables

Sample collection and measurements

Monte Carlo health risk assessment

Distribution and contamination of HMs

Text S1 Microwave digestion method of the HNO_3-HF-HCl system

Text S2 Interpretation of the applicability of the health risk assessment methods

Table S1 European Community Reference Bureau three-stage extraction program

Table S2 Element background value and toxicity response coefficient value table

Table S3 Evaluation index of sediment potential ecological harm

Table S4 Evaluation index of pollution by the chemical form of heavy metals in sediments

Table S5 Mean, standard deviation, range and distribution of the exposure and toxicological parameters used in the human health risk assessment

Table S6 Water quality parameters

Table S7 Critical effect concentration and possible effect concentration value table

Table S8 RAC value and risk level of heavy metals in soil

Table S9 Carcinogenic and non-carcinogenic risk index of heavy metals in surface sediments of Dongping Lake for children and adult men and women

Text S1 Microwave digestion method of the HNO_3-HF-HCl system

Take 0.2 g of sifted dry sediment sample and add 6 mL, 2 mL and 2 mL of HNO3, HF and HCl into the polytetrafluoroethylene digestion tank slowly in turn. Shake the tank slightly to make the sediment sample and acid fully mix and then start the microwave digestion. After digestion, the liquid sample is transferred to the crucible and washed with pure water to reduce the loss. Use an electric heating plate to heat at 170 degrees Celsius until thick droplets form, then remove and cool. The remaining sample in the crucible is filled with 1% nitric acid and mixed, left to settle overnight, and then filtered with 0.22 μm water filter membrane to be tested on the machine. An Agilent 7800 ICP-MS is used to determine the content of V (vanadium), Cr (chromium), Mn (manganese), Co (cobalt), Ni (nickel), Cu (copper), Zn (zinc), Cd (cadmium), and Pb (lead) in the water samples and sediment samples. In the process of heavy metal testing, for quality control, the recovery of heavy metal elements is 84.5%–104.1% by the tests of blank, sediment reference material (GBW07427) and parallel samples. The organic matter in sediments is measured using the burn loss method (LOI)[42], which can be determined by burning dry sediments at 550 degrees Celsius for four hours. The three-stage continuous extraction method of the European Community Reference Office (BCR) is applied for metal separation in the sediment samples. The BCR sequence extraction procedure is carried out by weighing 0.8 g from the sediment samples that have been screened with 200 mesh nylon. The metals in the sediment could be divided into four different components: acid-soluble, reducible, oxidizable and residual. The sequential chemical extraction procedure is shown in the Table S1. The determination method used for the content of each form is the same as that for the total

heavy metal.

Text S2 Interpretation of the applicability of the health risk assessment methods

The risk of heavy metals to human health is mainly affected by the environmental pollution level, human exposure pathway and exposure behavior of heavy metals in the study area. Most of the concentration changes of heavy metals in the human body can be explained by the concentration changes of heavy metals in environmental media[43].

According to the health risk model proposed by the EPA, the human health risk assessment method takes the health risk degree as an evaluation index and combines the pollution of heavy metals in the environment with human health, so as to quantitatively describe the health risks caused by the pollution of heavy metals in the environment. Heavy metals can pose a threat to human health through direct ingestion, oral and nasal inhalation, and skin absorption. However, in lake ecosystems, the most important way for lake-bottom surface sediment pollutants to enter the human body is direct ingestion through drinking water. As in other studies, when analyzing the risk of exposure to street dust for human health, considering the other two ingestion routes has little impact on the final result. This is because the correlation of the bioaccessibility of metals may not be included in the consideration of the other two pathways, which leads to an overestimation of the correlation of pathways[11,44,45]. Therefore, from the perspective of human protection, to achieve the purpose of simplifying the model, only the drinking water pathway is considered as the exposure pathway to calculate and evaluate the human health risks. The health risks of adult males, females and children were evaluated based on the population conditions and field survey results, and the parameter values in the model were adjusted appropriately. The U.S. Environmental

Protection Agency and the International Agency for Research on Cancer have integrated a database of risk information to classify heavy metals as carcinogens and non-carcinogens. In order to truly reflect the health risks of residents near the study area, the V, Cr, Mn, Co, Ni, Cu, Zn, Pb and Cd in 31 sediment samples collected in April 2023 were selected as non-carcinogenic factors. Among them, Cr, Pb and Cd have carcinogenicity at the same time and are calculated as carcinogenic factors.

Table S1. European Community Reference Bureau three-stage extraction program

Step	Fractions	Reagents	Operating conditions
1	Acid soluble	32 ml of 0.11 $mol*L^{-1}$ HAC solution	22℃ room temperature 16 h，constant agitation
2	Reducible	32 ml of 0.5 $mol*l^{-1}$ $NH_2OH·HCl$ (pH = 1.5 with HNO3) solution	First centrifuge the sample of the previous step 300 rmp for 20 min. Filter the supernatant with 0.45 μm aqueous filter membrane, add 15 mL pure water, shake for 15 min, add hydroxylamine hydrochloride solution, turn and shake for 16 h at 25℃
3	Oxidizable	(1) 8 ml of 30% w/v H_2O_2<br>(2) 8 ml of 30% w/v H_2O_2<br>(3) 40 ml of 1 mol $*l^{-1}$ NH_4AC solution	First add 8 ml of reagent (1), slowly add, intermittently shake at room temperature for 5 min for 1 h, then heat the water bath at 85℃, evaporate to the remaining 3 mL of solution for 1 h, then add the reagent (2), heat the water bath at 85℃, evaporate to the remaining 1ml of solution for 1 h. Add reagent (3) at room temperature, 16 h, constant agitation
4	Residual	$HF+HCl+HNO_3$	Digested in crucible

Table S2 Element background value and toxicity response coefficient value table

Element	Cd	Cu	Ni	Pb	Co	Cr	V	Mn	Zn
Background value C_n^i (mg/kg)	0.113	21	28	20	11.1	60.65	75	571	59
Toxicity coefficient T_r^i	30	5	5	5	5	2	2	1	1

Table S3 Evaluation index of sediment potential ecological harm

E_r^i	Grades of ecological risk for a single metal	RI	Grades of potential ecological risk to the environment	I_{geo}	Pollution degree
$E_r^i < 40$	Low risk	$RI < 150$	Low risk	$0 < I_{geo} \leq 1$	Light pollution
$40 \leq E_r^i < 80$	Moderate risk	$150 \leq RI < 300$	Moderate risk	$1 < I_{geo} \leq 2$	Moderate pollution
$80 \leq E_r^i < 160$	Considerable risk	$300 \leq RI < 600$	Considerable risk	$2 < I_{geo} \leq 3$	Moderate to heavy pollution
$160 \leq E_r^i < 320$	High risk	$RI \geq 600$	High risk	$3 < I_{geo} \leq 5$	Heavy pollution
$E_r^i \geq 320$	Very high risk			$I_{geo} \geq 5$	Extremely heavy pollution

Table S4 Evaluation index of pollution by the chemical form of heavy metals in sediments

RAC	Risk level
RAC≤1%	Low risk
1%＜RAC≤10%	Moderate risk
10%＜RAC≤30%	Considerable risk
30%＜RAC≤50%	High risk
RAC＞50%	Very high risk

Table S5 Mean, standard deviation, range and distribution of the exposure and toxicological parameters used in the human health risk assessment

Symbol	Physical meaning	Unit	Type	Value			Reference
				Adult males	Adult females	Children	
BW	Body weight	kg	Lognormal	67.55±8.72	57.59±8.03	21.7±7.0	IBGE 2010
IR	Sediment ingestion rate	$mg\cdot d^{-1}$	Lognormal	65±40	65±40	200±55	Carrizales et al. (2006)[46] Pengetal (2016)[47]
ED	Exposure duration	a	Deterministic	24	24	6	U.S. Environmental Protection Agency (U.S. EPA) (2001)[48-53]
EF	Exposure frequency	$d\cdot a^{-1}$	Triangular	345（180–365）	345（180–365）	345（180-365）	Smith (1994)[54]
AT	Averaging time	d	Deterministic	ED×365(non-carcinogenic) 70×365(carcinogenic)	ED×365(non-carcinogenic) 70×365(carcinogenic)	ED×365(non-carcinogenic) 70×365(carcinogenic)	U.S. Environmental Protection Agency (U.S. EPA) (2001)[48-53]
RfD_{ing}	Intake	mg·	Deterministic	7.00×10^{-3}	7.00×10^{-3}	$7.00\times10^{-}$	The Risk Assessment Information System

(V)	reference dose（V）	kg^{-1} day^{-1}	stic			3	(RAIS) (2020)[55]
RfD_{ing} (Cr)	Intake reference dose （Cr）	mg· kg^{-1} day^{-1}	Deterministic	3.00×10^{-3}	3.00×10^{-3}	3.00×10^{-3}	The Risk Assessment Information System (RAIS) (2020)[55]
RfD_{ing} (Mn)	Intake reference dose （Mn）	mg· kg^{-1} day^{-1}	Deterministic	4.60×10^{-2}	4.60×10^{-2}	4.60×10^{-2}	The Risk Assessment Information System (RAIS) (2020)[55]
RfD_{ing} (Co)	Intake reference dose （Co）	mg· kg^{-1} day^{-1}	Deterministic	2.00×10^{-2}	2.00×10^{-2}	2.00×10^{-2}	The Risk Assessment Information System (RAIS) (2020)[55]
RfD_{ing} (Ni)	Intake reference dose （Ni）	mg· kg^{-1} day^{-1}	Deterministic	2.00×10^{-2}	2.00×10^{-2}	2.00×10^{-2}	The Risk Assessment Information System (RAIS) (2020)[55]
RfD_{ing} (Cu)	Intake reference dose （Cu）	mg· kg^{-1} day^{-1}	Deterministic	4.00×10^{-2}	4.00×10^{-2}	4.00×10^{-2}	The Risk Assessment Information System (RAIS) (2020)[55]
RfD_{ing} (Zn)	Intake reference dose （Zn）	mg· kg^{-1} day^{-1}	Deterministic	3.00×10^{-1}	3.00×10^{-1}	3.00×10^{-1}	The Risk Assessment Information System (RAIS) (2020)[55]

$RfD_{ing(Cd)}$	Intake reference dose （Cd）	mg·kg^{-1} day^{-1}	Deterministic	1.00×10^{-3}	1.00×10^{-3}	1.00×10^{-3}	The Risk Assessment Information System (RAIS) (2020)[55]
$RfD_{ing(Pb)}$	Intake reference dose （Pb）	mg·kg^{-1} day^{-1}	Deterministic	3.50×10^{-3}	3.50×10^{-3}	3.50×10^{-3}	The Risk Assessment Information System (RAIS) (2020)[55]
$SF_{ing(Cr)}$	Slope factor for ingestion （Cr）	mg·kg^{-1} day^{-1}	Deterministic	5.0×10^{-1}	5.0×10^{-1}	5.0×10^{-1}	Chen et al. (2016)[56]
$SF_{ing(Pb)}$	Slope factor for ingestion（Pb）	mg·kg^{-1} day^{-1}	Deterministic	8.50×10^{-3}	8.50×10^{-3}	8.50×10^{-3}	Chen et al. (2016)[56]
$SF_{ing(Cd)}$	Slope factor for ingestion（Cd）	mg·kg^{-1} day^{-1}	Deterministic	6.30	6.30	6.30	Chen et al. (2016)[56]

Table S6 Water quality parameters

Element	T (°C)	TDS (mg/L)	Ec (μS)	DO (mg/L)	pH	NH_4^+-N (mg/L)	NO_2^-–N (mg/L)	TN (mg/L)	NO_3^--N	HPI
Sample size	33	33	33	33	33	33	33	33	33	33
Minimum	13.90	718	924	9.56	7.63	0.09	0.010	0.66	0.22	3.67
Maximum	21.80	1050	1428	12.24	8.15	0.89	0.12	4.94	4.37	19.04
Median	15.70	814	1158	10.64	7.98	0.17	0.016	1.89	1.55	5.01
Mean	15.82	820	1138.03	10.63	7.97	0.25	0.019	1.95	1.57	7.61
Standard deviation	1.61	57.17	95.24	0.58	0.11	0.17	0.020	0.81	0.73	4.72

Table S7 Critical effect concentration and possible effect concentration value table

Element	Cu	Ni	Pb	Cr	Zn
PEL	141	78.6	200	160.4	461
TEL	56.2	35.4	47.3	52.3	79.9

The TEL is the boundary line of the pollutant concentration to determine “with” or “without” obvious effect. When the pollutant concentration is lower than the TEL, it is considered that the pollutant poses no significant harm to aquatic organisms. The PEL is the threshold at which the hazard is considered to occur “frequently”, and when the concentration of the pollutant exceeds the PEL, it is considered that the pollutant frequently causes adverse effects in organisms.

Table S8 RAC value and risk level of heavy metals in soil

Element	RAC Mean/%	RAC≤1%		1%＜RAC≤10%		10%＜RAC≤30%		30%＜RAC≤50%		RAC＞50%	
		Amount	Percentage/%	Amount	Percentage/%	Amount	Percentage/%	Amount	Percentage/%	Amount	Percentage/%
V	0.04	31	100	0	0	0	0	0	0	0	0
Cr	0.014	31	100	0	0	0	0	0	0	0	0
Co	2.31	0	0	31	100	0	0	0	0	0	0
Ni	4.06	0	0	31	100	0	0	0	0	0	0
Cu	1.41	20	64.51	11	35.49	0	0	0	0	0	0
Zn	3.16	0	0	31	100	0	0	0	0	0	0
Cd	48.64	0	0	0	0	0	0	20	64.51	11	35.49
Pb	1.33	0	0	31	100	0	0	0	0	0	0
Risk level		Low risk		Moderate risk		Considerable risk		High risk		Very high risk	

Table S9 Carcinogenic and non-carcinogenic risk index of heavy metals in surface sediments of Dongping Lake for children and adult men and women

Element	Child		Male		Female	
	CRI	TEHI	CRI	TEHI	CRI	TEHI
Cd	1.33E-06	2.12E-03	1.03E-07	1.63E-04	1.18E-07	1.88E-04
Co		3.92E-03		2.88E-04		3.45E-04
Cr	3.30E-04	2.20E-01	1.58E-05	1.05E-02	1.87E-05	1.25E-02
Cu		3.54E-03		2.64E-04		2.99E-04
Mn		7.03E-02		5.03E-03		5.96E-03
Ni	3.01E-04	8.85E-03	2.28E-05	6.69E-04	2.59E-05	7.61E-04
Pb			3.98E-02	2.93E-03	3.37E-03	
V			5.83E-02	4.09E-03	4.90E-03	
Zn			1.10E-03	8.07E-05	9.54E-05	

Reference

[42] DEAN W E J J O S R. Determination of carbonate and organic matter in calcareous sediments and sedimentary rocks by loss on ignition; comparison with other methods [J]. 1974, 44(1): 242-8.

[43] CUI Y B, BAI L, LI C H, et al. Assessment of heavy metal contamination levels and health risks in environmental media in the northeast region [J]. Sust Cities Soc, 2022, 80: 15.

[44] XIAO-DONG W, WEI T, XUE-YAN Z. Distribution Characteristics and Health Ｒisk Assessment of Metal Elements for Groundwater in the Ningxia Ｒegion of China [J]. Environ Sci Technol, 2022, 43(01): 329-38.

[11] ZHANG Z F, YU N, ZHANG Y H, et al. Characteristics and source analysis of water pollution in dry season (November to March) of Dongping Lake (China) [J]. Agric Water Manage, 2022, 273: 10.

[45] ZHAO L, HONG Z, WEN-JIA1 C, et al. Seasonal Distribution Characteristics and Health Ｒisk Assessment of Heavy Metals in Surface Water of Qingjiang Ｒiver [J]. Environmental Science, 2021, 42(01): 175-83.

[46] CARRIZALES L, RAZO I, TéLLEZ-HERNáNDEZ J I, et al. Exposure to arsenic and lead of children living near a copper-smelter in San Luis Potosi, Mexico: Importance of soil contamination for exposure of children [J]. 2006, 101(1): 1-10.

[47] PENG C, CAI Y M, WANG T Y, et al. Regional probabilistic risk assessment of heavy metals in different environmental media and land uses: An urbanization-affected drinking water supply area [J]. Sci Rep, 2016, 6: 9.
[48] U.S. Environmental Protection Agency (US EPA), 1984. Health Effects Assessment for Barium. https://nepis.epa.gov/Exe/ZyPDF.cgi/2000FDFS.PDF?Dockey=2000FDFS.PDF. (Accessed 15 May 2021).
[49] U.S. Environmental Protection Agency (US EPA), 1987. Barium: Health Advisory. https://nepis.epa.gov/Exe/ZyPDF.cgi/94006C0D.PDF?Dockey=94006C0D.PDF. (Accessed 15 May 2021).
[50] U.S. Environmental Protection Agency (US EPA), 1989. Risk Assessment Guidance for Superfund Volume I: Human Health Evaluation Manual (Part A). https://www.epa. gov/sites/default/files/2015-09/documents/rags_a.pdf. (Accessed 1 May 2021).
[51] U.S. Environmental Protection Agency (US EPA), 2001. Risk Assessment Guidance for Superfund: Volume III– Part A, Process for Conducting Probabilistic Risk Assessment. https://www.epa.gov/sites/default/files/2015-09/documents/rags3adt_complete.pdf. (Accessed 1 May 2021).
[52] U.S. Environmental Protection Agency (US EPA), 2007. Method 6200: Field Portable X–ray Fluorescence Spectrometry for the Determination of Elemental Concentrations in Soils and Sediment. https://www.epa.gov/sites/default/files/2015-12/documents/6200.pdf. (Accessed 5 May 2018).
[53] U.S. Environmental Protection Agency (US EPA), 2017. Method 1340: In Vitro Bioaccessibi lity Assay for Lead in Soil. https://www.epa.gov/hw-sw846/sw-846-test-method-1340 vitro-bioaccessibility-assay-lead-soil. (Accessed 2 September 2022).
[54] SMITH R L J R A. Use of Monte Carlo simulation for human exposure assessment at a superfund site [J]. 1994, 14(4): 433-9.
[55] The Risk Assessment Information System (RAIS), 2020. RAIS Chemical PRG Calculator. https://rais.ornl.gov/cgi-bin/prg/PRG_search?select=chem. (Accessed 1 May 2020).
[56] CHEN H, TENG Y, LU S, et al. Source apportionment and health risk assessment of trace metals in surface soils of Beijing metropolitan, China [J]. 2016, 144: 1002-11.
